# Hippo Pathway Core Genes Based Prognostic Signature and Immune Infiltration Patterns in Lung Squamous Cell Carcinoma

**DOI:** 10.3389/fonc.2021.680918

**Published:** 2021-04-29

**Authors:** Chang Gu, Jiafei Chen, Xuening Dang, Chunji Chen, Zhenyu Huang, Weidong Shen, Xin Shi, Chenyang Dai, Chang Chen

**Affiliations:** ^1^ Department of Thoracic Surgery, Shanghai Pulmonary Hospital, Tongji University School of Medicine, Shanghai, China; ^2^ Department of Colorectal and Anal Surgery, Xinhua Hospital, Shanghai Jiao Tong University School of Medicine, Shanghai, China; ^3^ Shanghai Colorectal Cancer Research Center, Shanghai, China; ^4^ Department of Thoracic Surgery, Shanghai Chest Hospital, Shanghai Jiao Tong University, Shanghai, China; ^5^ Division of Functional Immunology, Institute for Genetic Medicine, Hokkaido University, Hokkaido, Japan; ^6^ Department of Cardiology, Shanghai Chest Hospital, Shanghai Jiao Tong University, Shanghai, China

**Keywords:** the Hippo pathway, WWC1, LATS2, SIGLEC15, CD274, lung squamous cell carcinoma

## Abstract

**Background:**

We investigated the prognostic effects and their patterns of immune infiltration of hippo pathway core genes in lung squamous cell carcinoma, in order to find some clues for underlying mechanisms of LUSC tumorigenesis and help developing new therapeutic methods.

**Methods:**

The mutational data, transcriptome data and corresponding clinical medical information of LUSC patients were extracted from The Cancer Genome Atlas (TCGA) database. Differential expression genes (DEGs) and Gene Ontology (GO), Kyoto Encyclopedia of Genes and Genomes (KEGG) analyses were explored. Survival analysis for the hippo core genes and the prognostic model were performed. Immune infiltration was estimated by CIBERSORT algorithm and some immune checkpoints-related genes were further investigated.

**Results:**

Overall, 551 LUSC samples were included in our study, consisting of 502 LUSC tumor samples and 49 adjacent normal samples, respectively. There were 1910 up-regulated DEGs and 2253 down-regulated DEGs were finally identified. The top five mutational hippo pathway core genes were LATS1 (4%), WWC1 (2%), TAOK1 (2%), TAOK3 (2%), and TAOK2 (2%), respectively. the mutation of LATS2 was highly associated with co-mutational NF2 (P <0.05) and TAOK1 (P <0.05). In survival analyses, we found only WWC1 (log-rank p = 0.046, HR = 1.32, 95% CI = 1–1.73) and LATS2 (log-rank p = 0.013, HR = 1.41, 95%CI = 1.08–1.86) had significant prognostic roles. After getting the three subgroups according to the subtyping results, we demonstrated that T cell gamma delta (p = 5.78e-6), B cell memory (p = 4.61e-4) and T cell CD4+ memory resting (p = 2.65e-5) had significant differences among the three groups. SIGLEC15 (P <0.01) and CD274 (P <0.05) also had statistical differences among the three subgroups.

**Conclusions:**

Our study verified the prognostic roles of WWC1 and LATS2 in LUSC patients. Immune checkpoints-related genes SIGLEC15 and CD274 had statistical differences among the three subgroups, which may provide new perceptions on the molecular mechanisms in LUSC and maybe helpful for precisely selecting specific LUSC patients with potential immunotherapy benefits.

## Introduction

Lung cancer remains the first lethiferous neoplasm all around the world (1–6). The outcomes of patients diagnosed with lung squamous cell carcinoma (LUSC) are significant poorer than those with lung adenocarcinoma (LUAD) (7, 8). Although the number of newly emerging LUSC patients is far less than that of LUAD patients, LUSC still poses threat to human health, given the huge population base (9). Besides, unlike LUAD, there has limited effective treatment for LUSC (10). Therefore, it is urgent to find and develop novel approaches for the treatment of LUSC.

The process of tumorigenesis, development and metastasis of lung cancer associates with dysregulation of many signaling pathways (11). Of the signaling pathways, the Hippo signaling pathway, considered as an evolutionarily conserved pathway, regulates cell differentiation and organ development *via* controlling the courses of cell proliferation and apoptosis (12). The core components of the Hippo signaling pathway are verified as a kinase cascade, which act directly on the primary downstream effectors, the yes-associated protein (YAP) and the transcriptional coactivator with PDZ-binding motif (TAZ) by phosphorylation, and thereby suppressing transcription process of downstream targeted genes (13). Recently, it has been proved that the dysregulation of the Hippo signaling pathway lead to tumorigenesis, development and metastasis of diverse tumors, including lung adenocarcinoma (14), breast cancer (15), hepatocellular carcinoma (16), gastric cancer (17) and so on. However, fewer studies focus on the effects of the Hippo signaling pathway on LUSC. Besides, Wang et al. (18) highlighted the significance of Hippo signaling, including 18 core genes, in LUSC through comprehensive molecular features. Therefore, it is important to further explore the underlining mechanisms of hippo pathway core genes in oncogenesis and progression of LUSC.

In recent years, cancer immunotherapy has been confirmed and regarded as a vitally important treatment alternative for cancer patients (19). With the clinical success of immune checkpoint blockade and chimeric antigen receptor T (CAR-T) cell therapies, a turning point has been reached in the field of cancer immunotherapy (20). Given the low targeted-effective-mutation frequency in LUSC, targeted therapy is not effective enough for LUSC patients while cancer immunotherapy provides improved treatment options for LUSC patients. In this study, we investigated the prognostic effects and their patterns of immune infiltration of hippo pathway core genes in lung squamous cell carcinoma, in order to find some clues for underlying mechanisms of LUSC tumorigenesis and help developing new therapeutic methods.

## Materials and Methods

### Data Acquisition

The mutational data, RNA-seq transcriptome data and corresponding clinical medical information of LUSC patients were extracted from The Cancer Genome Atlas (TCGA) database (https://portal.gdc.cancer.gov/). Overall, 551 LUSC samples were included in our study, consisting of 502 LUSC tumor samples and 49 adjacent normal samples, respectively.

### Selecting Differential Expression Genes (DEGs) and Gene Ontology (GO), Kyoto Encyclopedia of Genes and Genomes (KEGG) Analyses

The differential expression of mRNAs was obtained by “Limma” package. An adjusted-P value was used for reducing the false positive rate. We defined DEGs as adjusted-P value less than 0.05 and the absolute value of log(Fold Change) more than 1. Then, the DEGs’ corresponding GO and KEGG pathways were further analyzed using “ClusterProfiler” package for acquiring the potential targets and enriched pathways (21–23).

### Mutational Analysis

There are 18 hippo pathway core genes were identified, including NF2, WWC1, TAOK1-2, TAOK3, FRMD6, SAV1, STK-4, MOB1A/B, LATS1-2, YAP1, TAZ, and TEAD1-4 (18). The somatic mutations of 18 hippo pathway core genes, co-occurrence patterns (by Fisher’s exact test), oncogenic pathways in LUSC patients were identified and visualized by maftools package.

### Correlation Analysis

Spearman’s correlation analysis was utilized for describing the correlation between quantitative variables without a normal distribution. Multi-gene correlation analysis were performed and visualized by heatmap. P <0.05 was defined having statistical significance.

### Survival Analysis and the Development of Prognostic Model

All the mRNA expressions of 18 hippo pathway core genes were calculated and patients were separated by the median expression level of each gene (high expressed group and low express group). The Kaplan–Meier (KM) survival analyses were used to compare the survival difference between low and high expressed groups based on each hippo pathway core gene group, with log-rank test. Then, the univariate and multivariate Cox analyses were performed to predict the prognostic significance for overall survival (OS). After obtaining prognostic hippo pathway core genes in multivariate Cox analysis, all the prognostic genes and relevant clinical characteristics were included for the nomogram modeling, with 1, 2, 3, 5 survival prediction scores. Then, the calibration curves for each year were also drawn.

R package “ConsensusClusterPlus” was used for consistency analysis and the maximum number of clusters is 6. Besides, four fifth of the total sample is drawn 100 times, clusterAlg = “hc,” innerLinkage = “ward.D2.” Clustering heatmaps were then drawn by R package “pheatmap.” The gene expression heatmap retains genes with SD > 0.1. If the number of input genes is more than 1000, it will extract the top 25% genes after sorting the SD.

### Analysis of Immune Infiltration and Immune Checkpoints-Related Gene Expression

We estimated immune infiltration by The “Cell type Identification By Estimating Relative Subsets Of RNA Transcripts (CIBERSORT)” algorithm, which provides an estimation of the abundances of member cell types in a mixed cell population (24). The visualization of the results were realized by “ggplot2” and “pheatmap” packages in R software. We selected some immune checkpoints-related genes for analysis, containing CD274, CTLA4, HAVCR2, LAG3, PDCD1, PDCD1LG2, TIGIT, and SIGLEC15. And the expression of the eight genes in different LUSC groups were studied.

## Results

**Figure 1:**
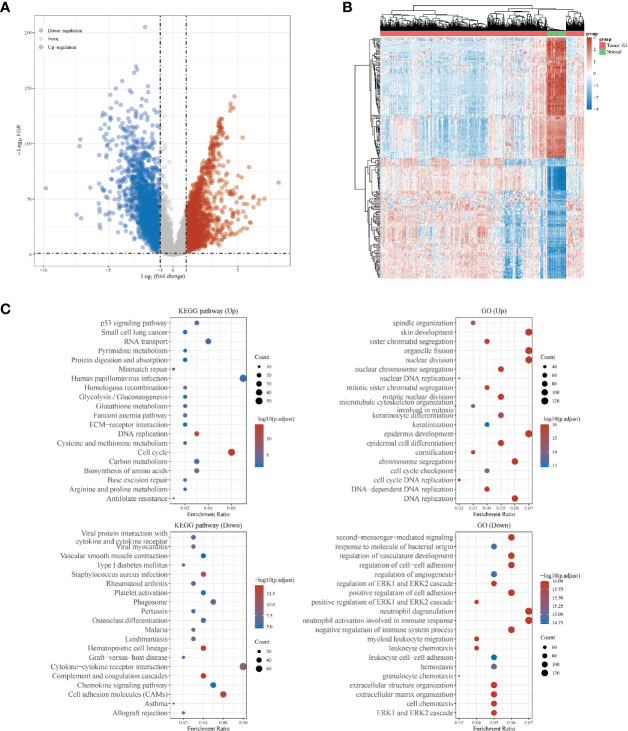
Differentially expressed genes (DEGs) in patients with lung squamous cell carcinoma (LUSC). **(A)** DEGs between tumor and normal tissues; **(B)** heatmap for DEGs in all the LUSC samples; **(C)** the Gene Ontology (GO) and Kyoto Encyclopedia of Genes and Genomes (KEGG) analyses for up- and down-regulated DEGs, respectively.

Overall, 551 LUSC samples were included in our study, consisting of 502 LUSC tumor samples and 49 adjacent normal samples, respectively. There were 1910 up-regulated DEGs and 2253down-regulated DEGs were finally identified. The volcano plot (**Figure 1A**) and heatmap (**Figure 1B**) of DEGs were constructed. The GO and KEGG analyses were further used for up-regulated and down-regulated DEGs, respectively. The top three KEGG pathways in up-regulated DEGs were human papillomavirus infection, cell cycle and RNA transport, respectively. While the top three KEGG pathways in down-regulated DEGs were cytokine–cytokine receptor interaction, cell adhesion molecules (CAMs) and phagosome, respectively. In GO analyses, the top three GO pathways in up-regulated DEGs were skin development, nuclear division and organelle fission, respectively. While the top three GO pathways in down-regulated DEGs were neutrophil degranulation, neutrophil activation involved in immune response and second-messenger-mediated signaling, respectively (**Figure 1C**).

**Figure 2:**
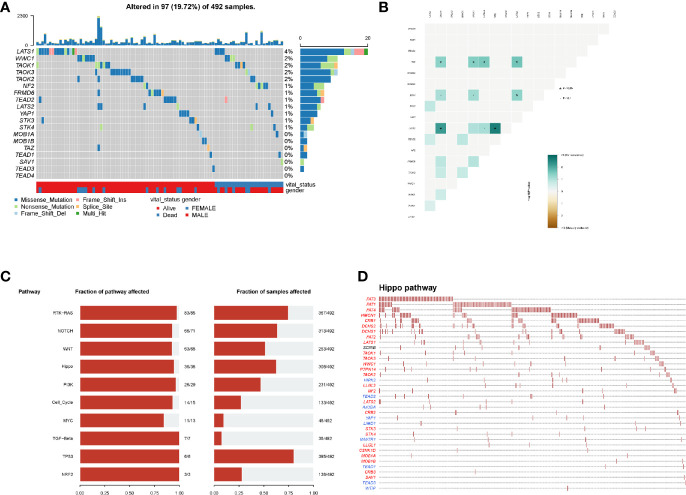
Mutation patterns of LUSC patients. **(A)** Oncoplot displays the mutational patterns of 18 hippo core genes in 97 LUSC patients; **(B)** the co-expression patterns of 18 hippo core genes in LUSC patients; **(C)** the enriched oncogenic pathways; **(D)** the hippo pathway in enriched oncogenic pathways.

In mutational analyses, 97 (19.72%) patients contained the mutational hippo pathway core genes in 492 LUSC patients. The top five mutational hippo pathway core genes were LATS1 (4%), WWC1 (2%), TAOK1 (2%), TAOK3 (2%) and TAOK2 (2%), respectively (**Figure 2A**). The co-occurrence patterns of the 18 hippo pathway core genes were then analyzed and we found the mutation of LATS2 was highly associated with co-mutational NF2 (P <0.05) and TAOK1 (P <0.05) (**Figure 2B**). Moreover, the oncogenic pathways were enriched (**Figure 2C** **)** and we further displayed the hippo pathway in oncogenic pathways (**Figure 2D** **)**.

**Figure 3:**
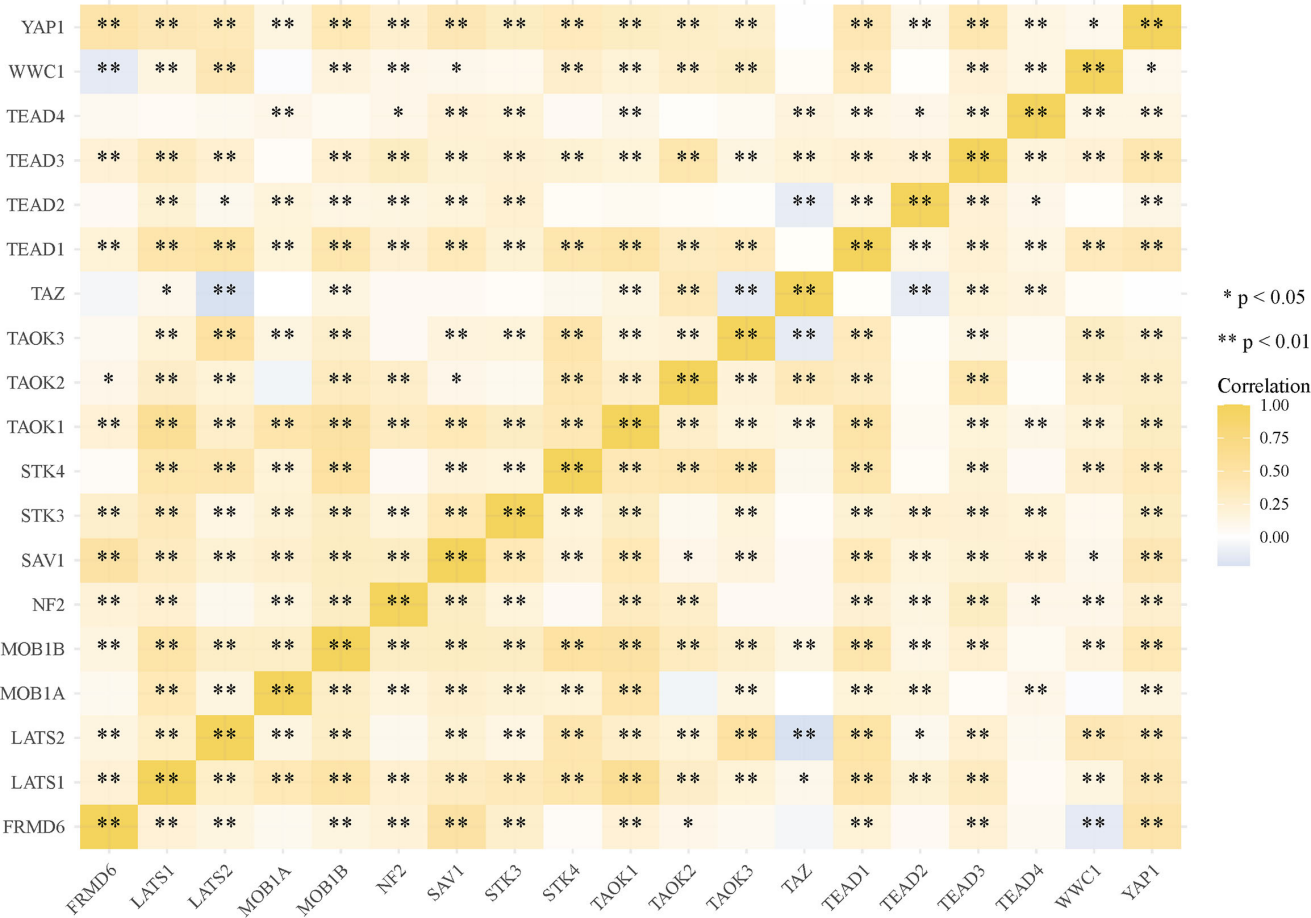
Correlation analysis among 18 hippo core genes in LUSC patients.

**Figure 4:**
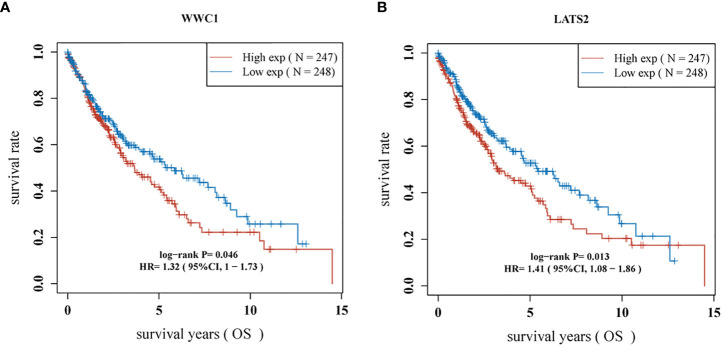
Survival curves according to the expression of **(A)** WWC1; **(B)** LATS2.

**Figure 5:**
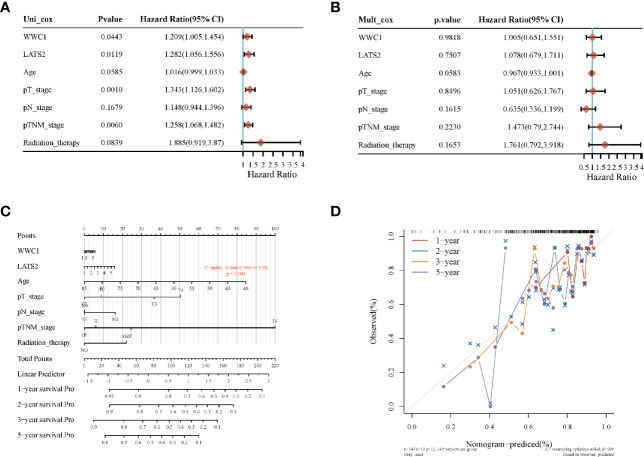
The identification of prognostic factor for OS and the development of nomogram. **(A)** Univariate Cox analysis; **(B)** multivariate Cox analysis; **(C)** nomogram for OS in LUSC patients; **(D)** the calibration curves for each year.

Most of the 18 hippo pathway core genes had positive correlations while four pairs had negative correlations in LUSC samples (**Figure 3**). Then, the survival analyses were performed, we found only WWC1 (log-rank p = 0.046, HR = 1.32, 95%CI = 1–1.73) (**Figure 4A**) and LATS2 (log-rank p = 0.013, HR = 1.41, 95%CI = 1.08–1.86) had significant prognostic roles in TCGA database (**Figure 4B**). Afterward, WWC1, LATS2, and related clinical factors were all included in Cox analyses. In univariate Cox proportional hazards regression, WWC1, LATS2, pT stage and pTNM stage were all significant independent prognostic factors for overall survival (OS). We built a nomogram for LUSC patients, with the c index as 0.644 (p <0.001, 95%CI = 0.569–0.719) (**Figure 5**).

**Figure 6:**
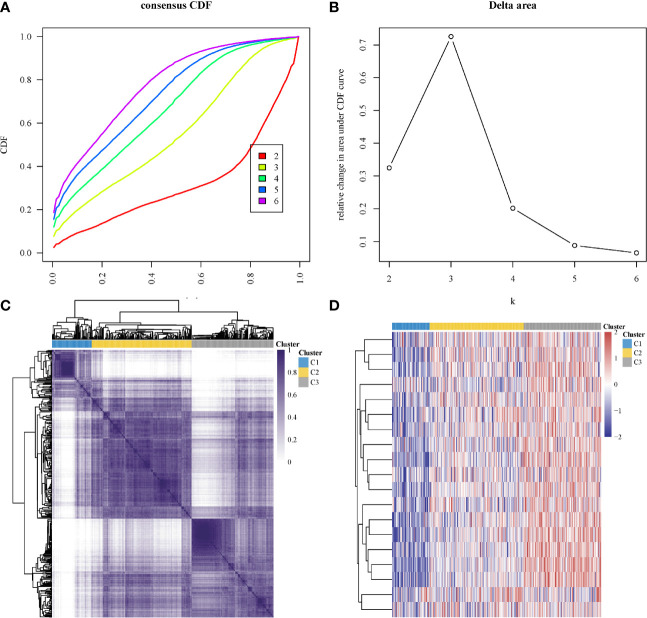
Identification of consensus clusters according to the expression similarity of hippo core genes. **(A)** Cumulative distribution function (CDF)(k = 2–6); **(B)** Relative change in area under CDF curve (k = 2–6); **(C)** the matrix of consensus clustering (k = 3); **(D)** heatmap of m6A-related gene expression in different subgroups, red represents high expression while blue represents low expression.

**Figure 7:**
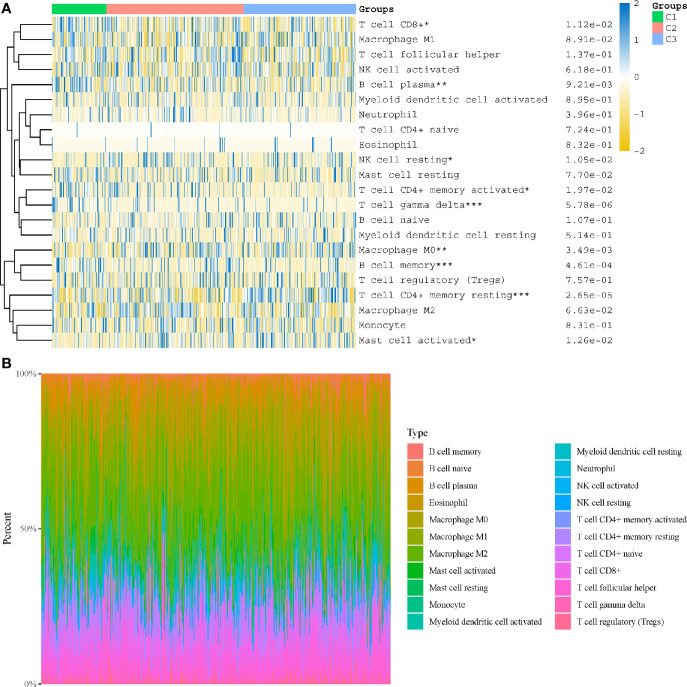
Immune infiltration estimated by CIBERSORT algorithm. **(A)** Immune cell score heat map; **(B)** the proportions of 22 types of immune cells were shown for each LUSC patient by a histogram. *p < 0.05, **p < 0.01, ***p < 0.001.

**Figure 8:**
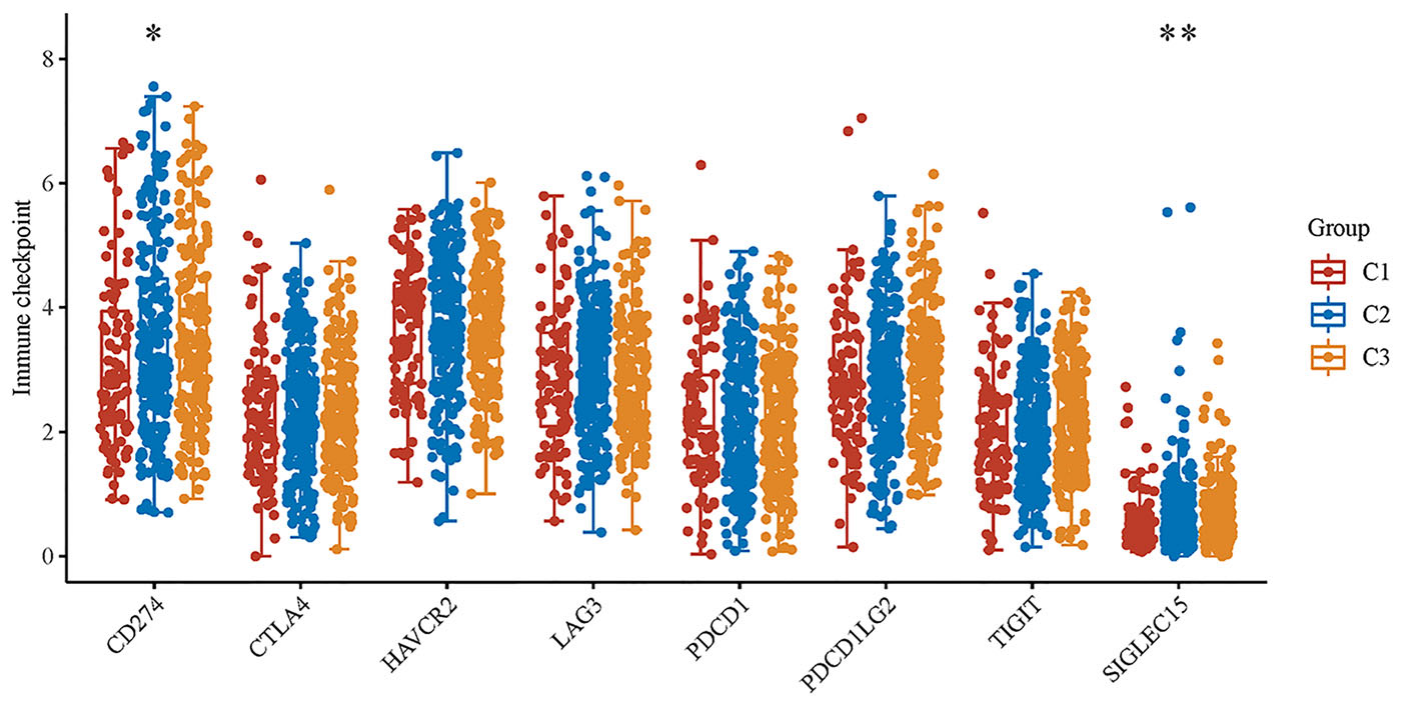
The expression distributions of 8 immune checkpoints-related genes in LUSC subgroups. *p < 0.05, **p < 0.01.

In subtyping analyses, we selected k = 3 as the cutoff value to develop subtyping groups (**Figure 6**). After getting the three subgroups according to the subtyping results, the immune infiltration of the three groups were compared by CIBERSORT algorithm. We demonstrated that T cell gamma delta (p = 5.78e-6), B cell memory (p = 4.61e-4) and T cell CD4+ memory resting (p = 2.65e-5) had significant differences among the three groups (**Figure 7A**). Besides, the proportions of 22 types of immune cells were shown for each LUSC patient by a histogram (**Figure 7B**). At last, we explored the expressed differences based on 8 immune checkpoints-related genes, which indicated that SIGLEC15 (P <0.01) and CD274 (P <0.05) had statistical differences among the three subgroups (**Figure 8**).

## Discussion

Although many novel biomarkers and prognostic models for lung cancer have been validated and applied, there still have limited effective treatment strategy for lung squamous cell carcinoma (10, 25–27). In this study, we explored the 18 hippo pathway core genes in LUSC cohort and demonstrated WWC1 and LATS2 had significant prognostic roles and a nomogram has been developed based on the above two genes and relevant clinical factors. After subtyping, there has significant differences in the immune infiltration among the three subgroups and CD274 and SIGLEC15 may acted as potential immunotherapeutic targets for high-risk LUSC patients.

In survival analyses, WWC1 (log-rank p = 0.046, HR = 1.32, 95%CI = 1–1.73) and LATS2 (log-rank p = 0.013, HR = 1.41, 95%CI = 1.08–1.86) had significant prognostic roles. WWC1, WW and C2 domain containing 1 (also named KIBRA), binds with NF2 and helps NF2 to regulate LATS1/2 *in vitro* (28). Besides, WWC1 has been proved to have vital role in various cancers. Knight et al. (29) identified WWC1 as a primary factor leading to the effects of 5q loss on the processes of growth and metastasis in triple-negative breast cancer (TNBC). Moleirinho et al. (30) demonstrated that WWC1 deficiency showed epithelial-to-mesenchymal transition (EMT) features, with decreased phosphorylation of YAP and LATS. In addition, they also found low WWC1 expression was associated with poor outcomes in primary breast cancer. In our study, the high expression of WWC1 displayed a worse prognosis when compared with those with low WWC1 expression in LUSC patients. LATS2, large tumor suppressor kinase 2, phosphorylates and inactivates YAP/TAZ (31). In non-small cell lung cancer (NSCLC) cells, the expression and transcription of LATS2 was suppressed by long non-coding RNA AGAP2-AS1, thereby inhibited NSCLC development and progression (32). Similar results was obtained by another research, 73 NSCLC and 22 normal tissues were collected and immunohistochemistry was used for further analysis. They found LATS2 acted as an independent prognostic factor for NSCLC patients and the higher expressed group had significant better survival (33). However, in this study, we found an opposite result, that is patients with high LATS2 expression had worse OS. The reasons would be the following: (1) the objects of the above two researches were all NSCLCs, but in our study, only LUSC cohort was enrolled. LUAD accounted for the majority of NSCLC and the high expression of LATS2 acted as a protective factor for OS in LUAD patients (log-rank p = 3.9e-9, HR = 0.47, 95%CI = 0.36–0.6. Data obtained by K-M plotter and it was not shown). The effects of LATS2 in LUAD may neutralize that in LUSC when the whole NSCLC patients were included; (2) a great heterogeneity is existed in lung cancer, especially in advanced-stage lung cancer, which may cause some bias.

After getting the three subgroups according to the subtyping results, the immune infiltration of the three groups were compared by CIBERSORT algorithm. We demonstrated that T cell gamma delta (p = 5.78e-6), B cell memory (p = 4.61e-4) and T cell CD4+ memory resting (p = 2.65e-5) had significant differences among the three groups. The above results meant the three subgroups divided by 18 hippo pathway core genes had good ability to distinguish patients with different immune status. Furthermore, it would be helpful for precise selection of LUSC patients who need immunotherapy. We also explored the expressed differences based on 8 immune checkpoints-related genes, which indicated that SIGLEC15 (P <0.01) and CD274 (P <0.05) had statistical differences among the three subgroups. CD274 (ligand programmed death-ligand 1, PD-L1) can negatively modulate T-cell antitumor activity *via* binding to the PD-1 receptor on the T cells (34). Therefore, anti-PD-1 therapy has an extensive use in almost all types of cancer. It has been verified that PD-L1 expression was associated with T-cell infiltration (35). In our study, we found a synchronous significant differences in PD-L1 expression and T-cell infiltration among the three subtypes, which was similar to previous studies. Overexpression of PD-L1 in tumor microenvironment is the main immune evasion mechanism in some cancer patients. SIGLEC15, acted as an immune suppressor, inhibits antigen-specific T cell responses *in vitro* and *in vivo* and its expression was mutually exclusive to PD-L1, which means SIGLEC15 could be a promising target in patients with noneffective PD-L1 therapy (36).

There are still some limitations in this study. First, although the data of LUSC patients were extracted from TCGA, the number of patients is still limited; second, all the patients were from Western populations and the molecular features of Eastern populations are still unknown. Third, there is no validation by our own LUSC cohort.

In conclusions, our study verified the prognostic roles of WWC1 and LATS2 in LUSC patients. After subtyping by hippo core genes, T cell gamma delta, B cell memory and T cell CD4+ memory resting had significant differences among the three subgroups. Immune checkpoints-related genes SIGLEC15 and CD274 had statistical differences among the three subgroups, which may provide new perceptions on the molecular mechanisms in LUSC and maybe helpful for precisely selecting specific LUSC patients with potential immunotherapy benefits.

## Data Availability Statement

The datasets presented in this study can be found in online repositories. The names of the repository/repositories and accession number(s) can be found in the article/**Supplementary Material**.

## Author Contributions

Conception and design: CG, CC, CD, and XS. Administrative support: CC and CD. Collection and assembly of data: CG, JC, ZH, and CJC. Data analysis and interpretation: CG, JC, ZH, and CJC. Manuscript writing: all authors. All authors contributed to the article and approved the submitted version.

## Funding

This work was funded by the National Natural Science Foundation of China (81900280) and Shanghai Sailing Program (19YF1431600), the National Natural Science Foundation of China (81802256), the “Chen Guang” project supported by Shanghai Municipal Education Commission and Shanghai Education Development Foundation (18CG19) and the “Outstanding Young Talent” project supported by Shanghai Pulmonary Hospital (FKYQ1907), Shanghai Rising Star Program (20QA1408300), Clinical Research Plan of Shanghai Hospital Development Center (SHDC2020CR4028, SHDC2020CR1021B), National Key Research and Development Project (2019YFE0101200), Fundamental Research Funds for the Central Universities (22120180607), Shanghai Science and Technology Committee (20YF1441100 & 20XD1403000 & 18DZ2293400), Shanghai Municipal Health Commission (2019SY072 & 2018ZHYL0102), Shanghai Pulmonary Hospital Innovation group project (Shanghai pulmonary hospital Innovation group project–“Chang Chen”) the Clinical Research Project of Shanghai Pulmonary Hospital (FK18001 & FK1904 & FKGG1805 & FK1936 & FK1943 & FKLY20007 & FKCX1906), Clinical Research Foundation of Shanghai Pulmonary Hospital (FK1944), and the “Outstanding young talent” project supported by Shanghai Pulmonary Hospital (FKYQ1907).

## Conflict of Interest

The authors declare that the research was conducted in the absence of any commercial or financial relationships that could be construed as a potential conflict of interest.

## Supplementary Material

Supplementary Table 1 The gene matrix of LUSC patients in TCGA database.
